# Supplementary material for: Spectroscopic Benchmarks by Machine Learning as Discriminant Analysis for Unconventional Italian Pictorialism Photography
Source: Polymers (Basel). 2024 Jun 28;16(13):1850. doi: 10.3390/polym16131850 (PMC11244078; doi:10.3390/polym16131850)
Supplement: Supplementary file 1 [file polymers-16-01850-s001.zip › polymers-3017631-supplementary materials.pdf]

polymers

MDPI

Supplementary Materials

Spectroscopic Benchmarks by Machine Learning as Discriminant Analysis for Unconventional Italian Pictorialism Photography

Claudia Scatigno [1,*], Lorenzo Teodonio [1], Eugenia Di Rocco [2] and Giulia Festa [1,*]

[1] CREF–Museo Storico della Fisica e Centro Studi e Ricerche Enrico Fermi, Via Panisperna 89a c/o P.za del Viminale 1, 00184 Roma, Italy
[2] Freelance Restorer CRAF–Centro di Ricerca e Archiviazione della Fotografia, Piazza Castello, 33097 Spilimbergo, Italy

* Correspondence: claudia.scatigno@cref.it (C.S.); giulia.festa@cref.it (G.F.)

Inventory number: FFPV_0001

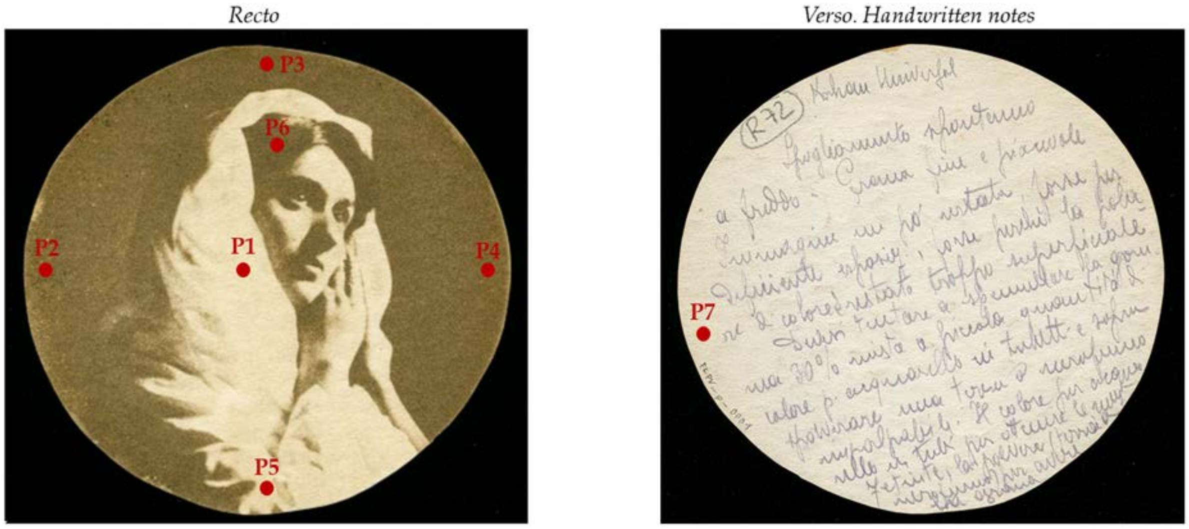

Figure S1. Recto and verso of photograph with the inventory number: FFPV_0001.

Inventory number: FFPV_0002

Recto | *Verso. Handwritten notes*

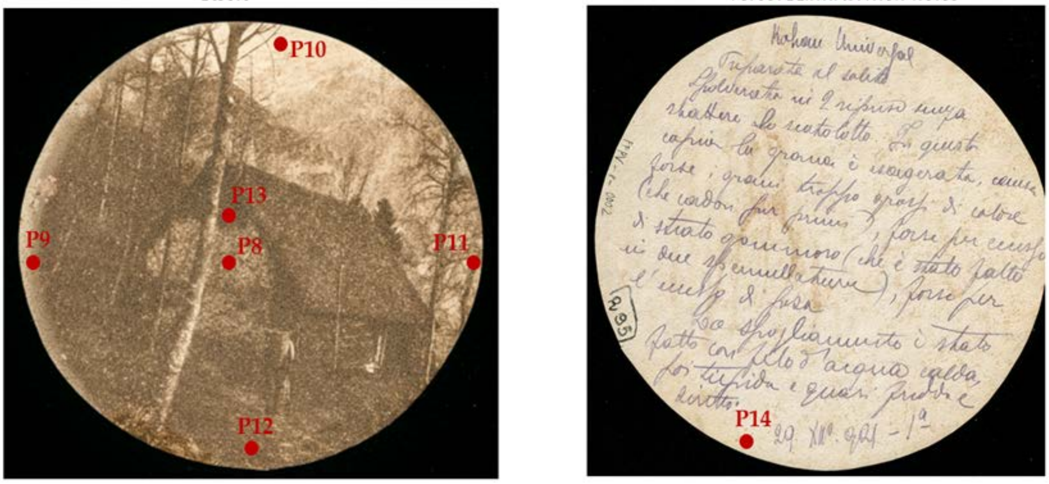

Figure S2. Recto and verso of photograph with the inventory number: FFPV_0002.

Inventory number: FFPV_0003

Recto | *Verso. Handwritten notes*

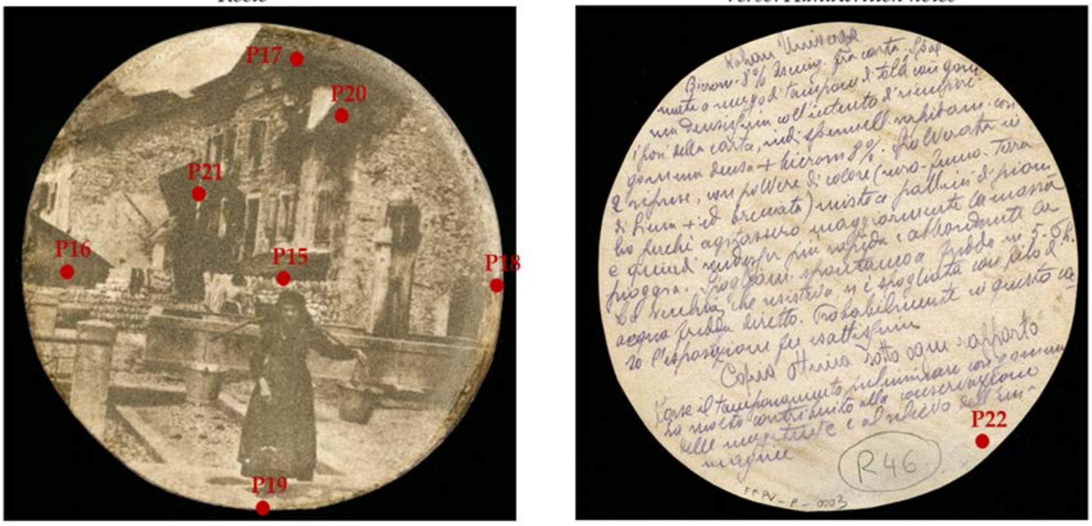

Figure S3. Recto and verso of photograph with the inventory number: FFPV_0003.

Inventory number: FFPV_0004

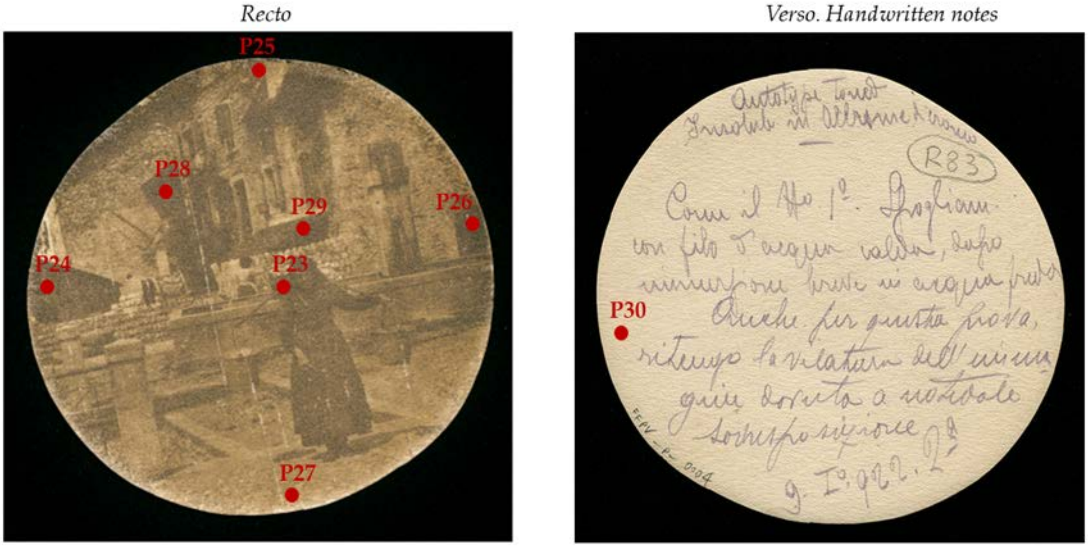

Figure S4. Recto and verso of photograph with the inventory number: FFPV_0004.

Inventory number: FFPV_0005

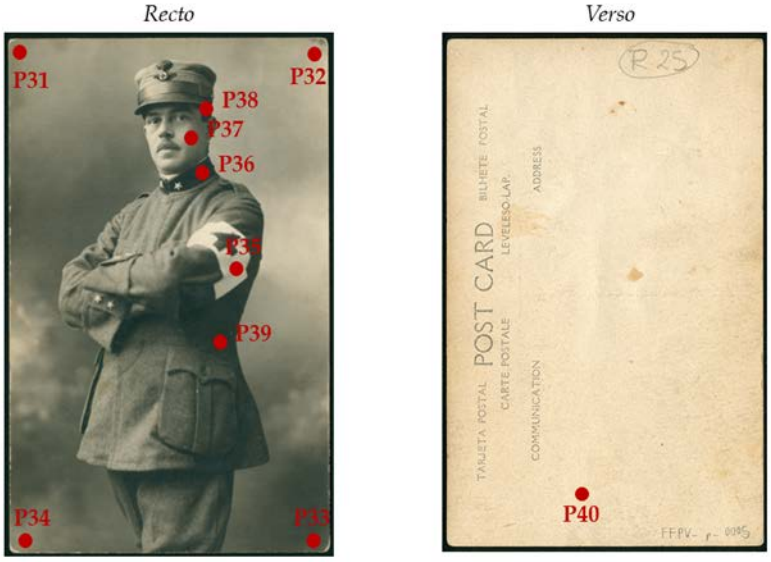

Figure S5. Recto and verso of photograph with the inventory number: FFPV_0005.

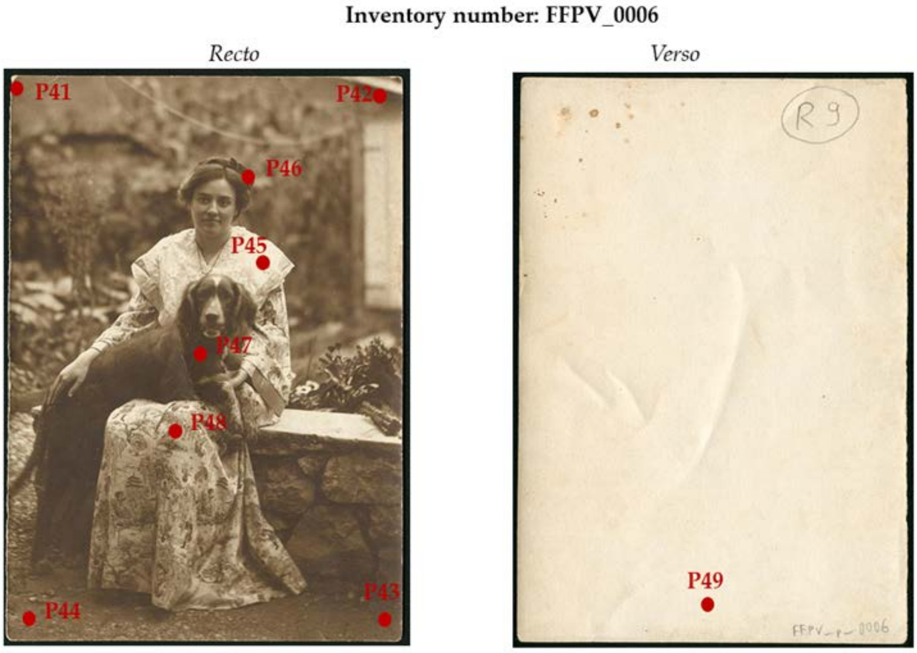

Figure S6. Recto and verso of photograph with the inventory number: FFPV_0006.

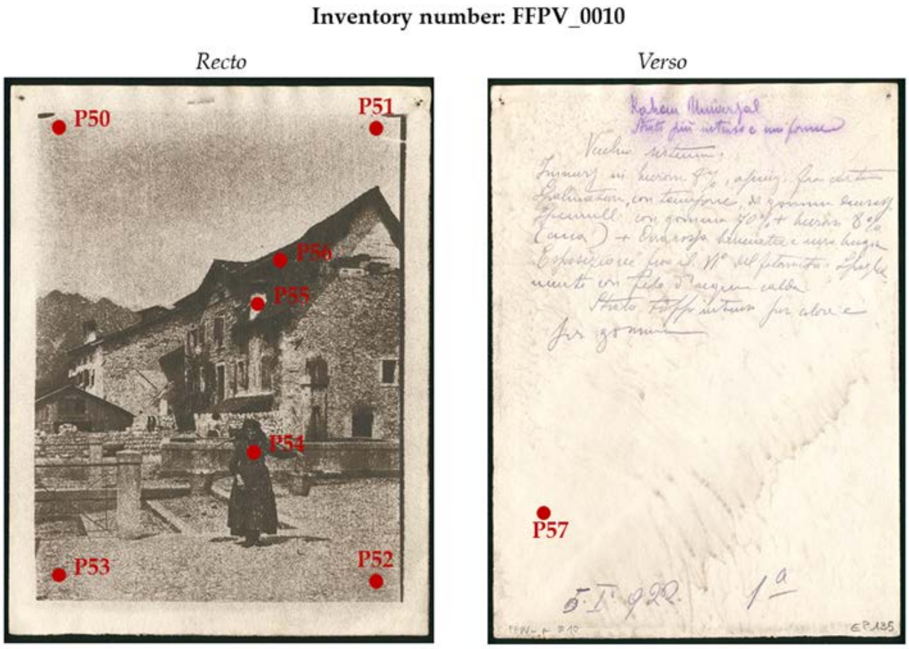

Figure S7. Recto and verso of photograph with the inventory number: FFPV_0010.

Inventory number: FFPV_0011

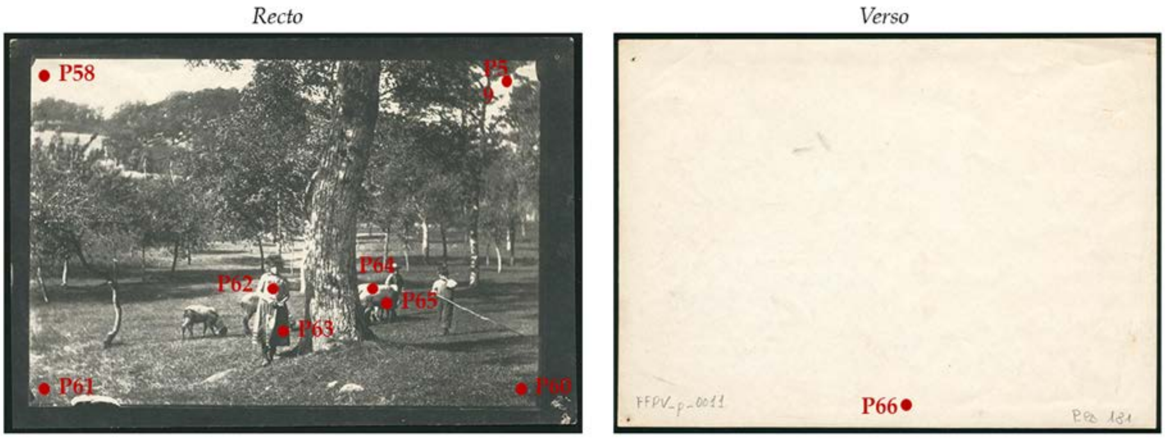

Figure S8. Recto and verso of photograph with the inventory number: FFPV_0011.

Inventory number: FFPV_0018

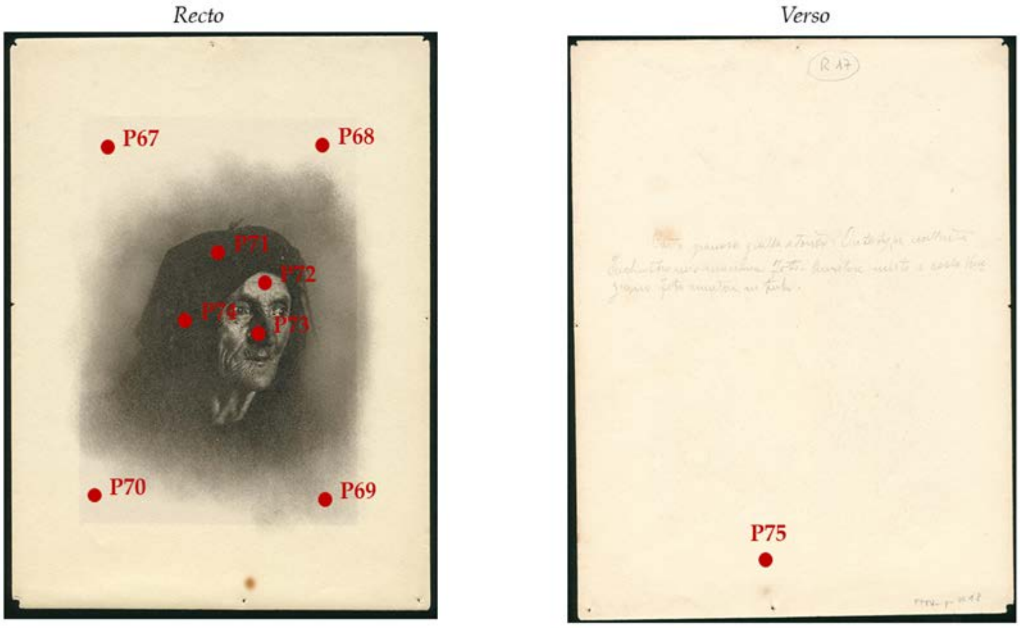

Figure S9. Recto and verso of photograph with the inventory number: FFPV_0011.

Inventory number: FFPV_0019

Recto *Verso*

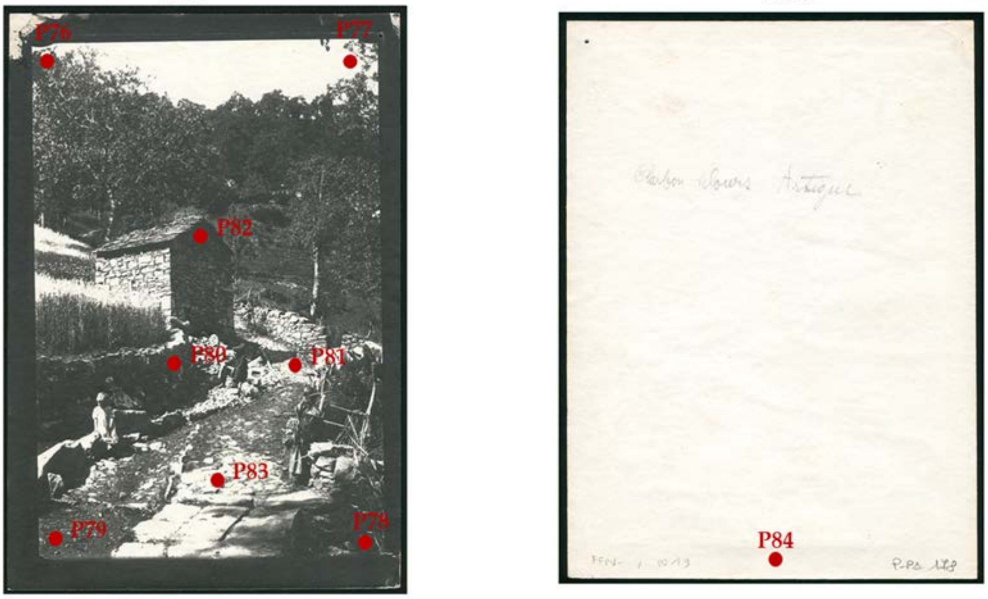

Figure S10. Recto and verso of photograph with the inventory number: FFPV_0019.

Inventory number: FFPV_0027

Recto *Verso*

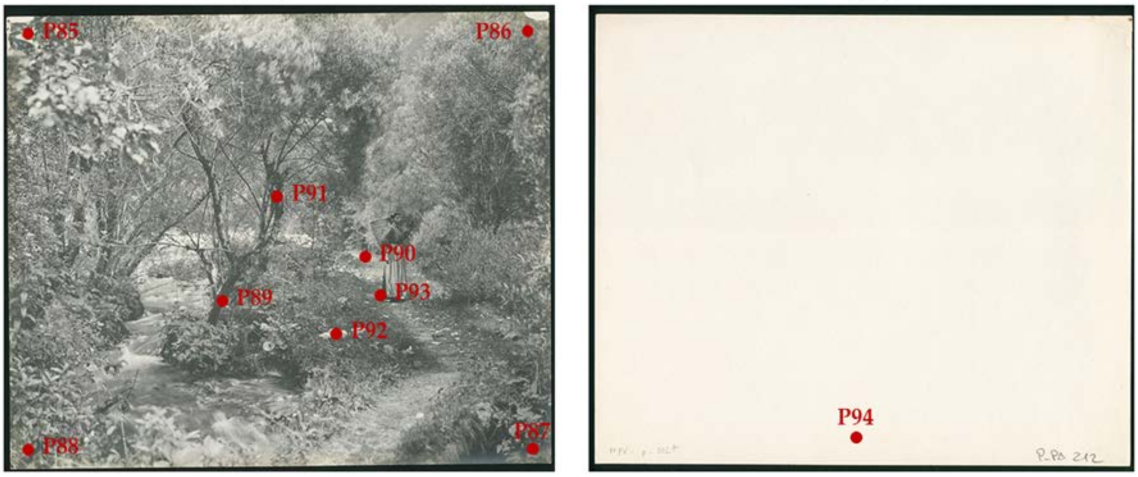

Figure S11. Recto and verso of photograph with the inventory number: FFPV_0027.

Inventory number: FFPV_0029

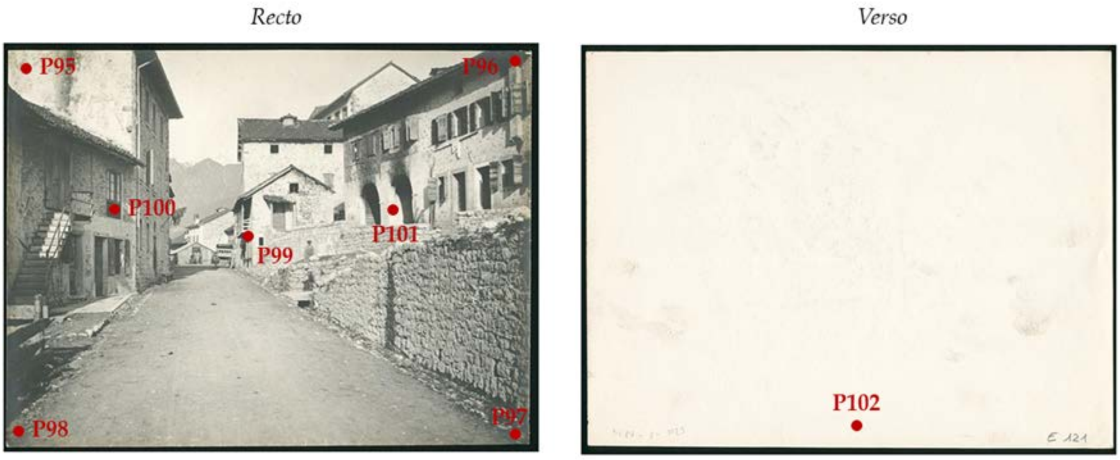

Figure S12. Recto and verso of photograph with the inventory number: FFPV_0029.

Inventory number: FFPV_p_0030

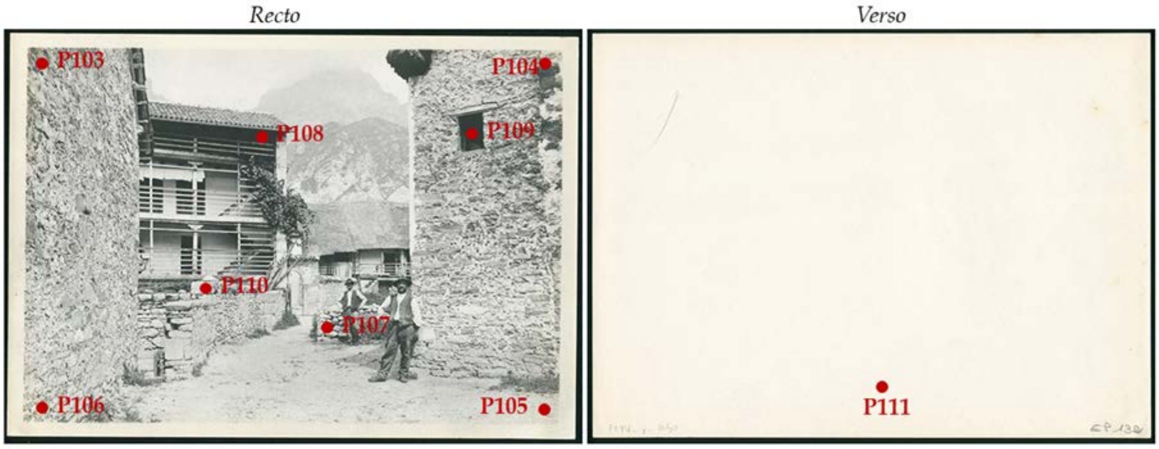

Figure S13. Recto and verso of photograph with the inventory number: FFPV_0030.

Inventory number: FFPV_0040

Recto *Verso*

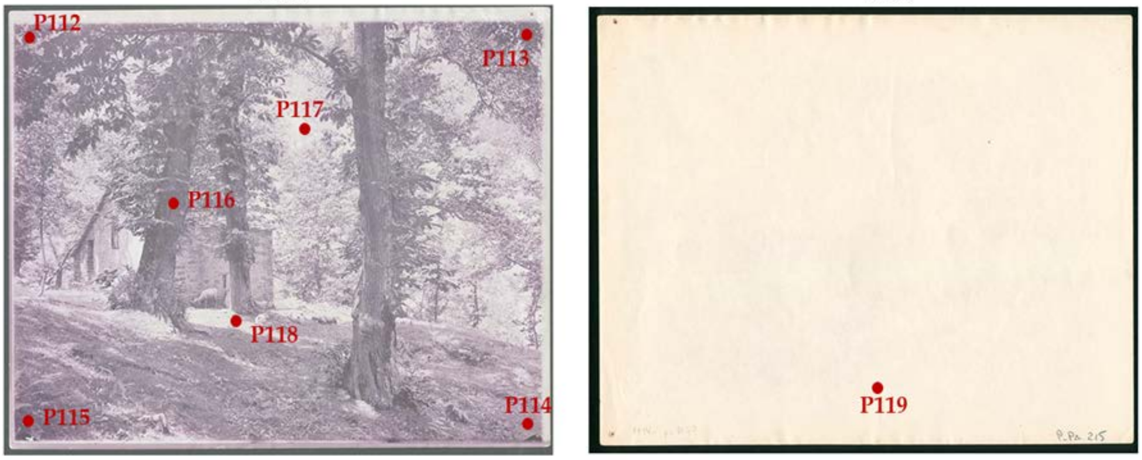

Figure S14. Recto and verso of photograph with the inventory number: FFPV_0040.

Inventory number: FFPV_0041

Recto *Verso*

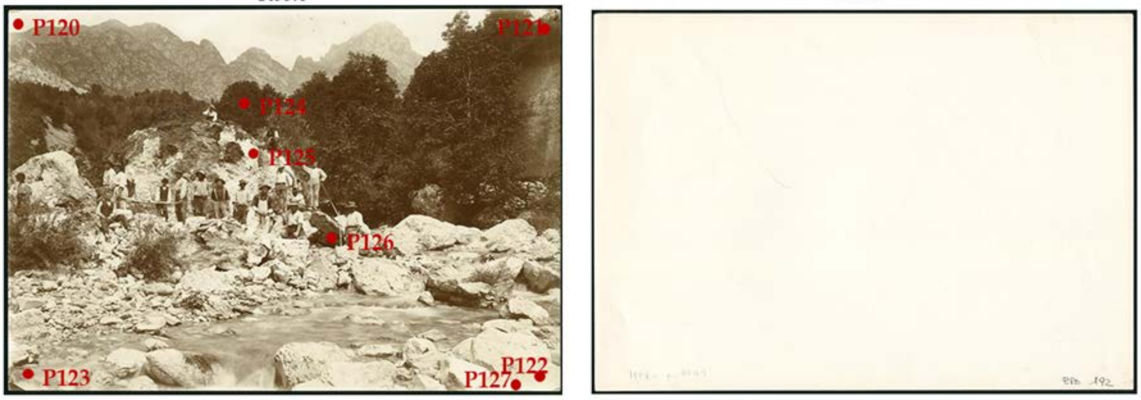

Figure S15. Recto and verso of photograph with the inventory number: FFPV_0041.

Inventory number: FFPV_0047

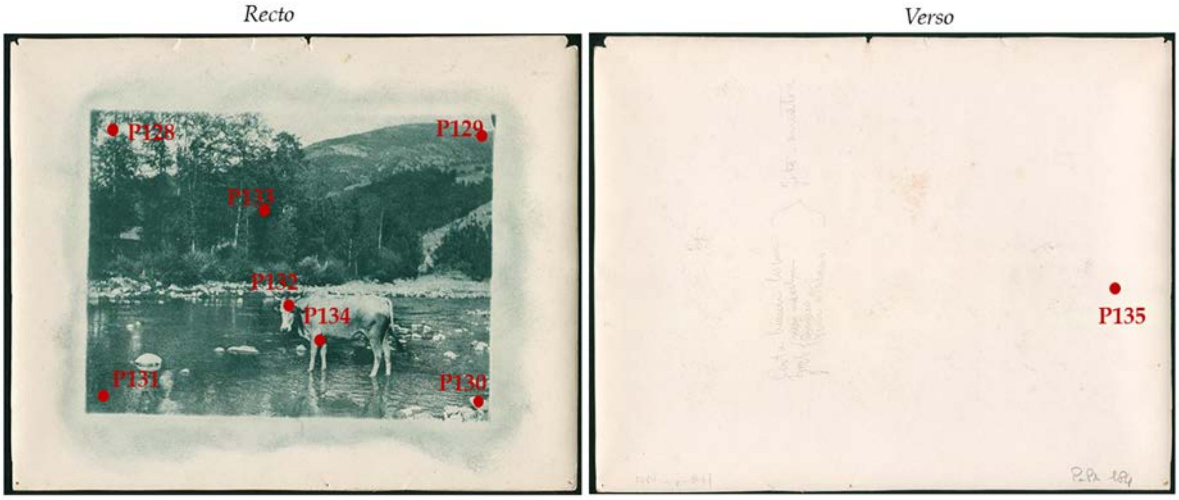

Figure S16. Recto and verso of photograph with the inventory number: FFPV_0047.

Inventory number: FFPV_0052

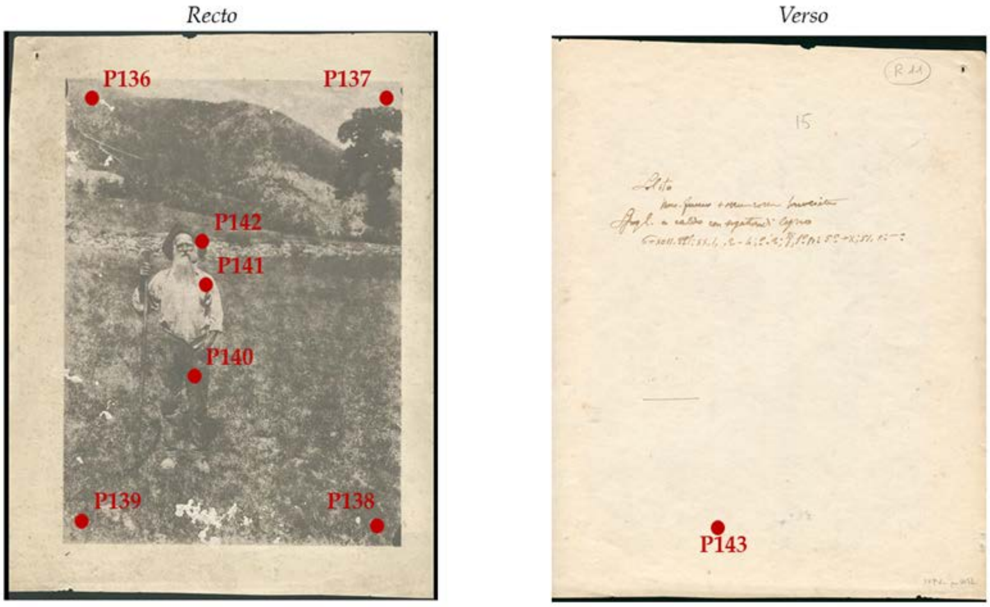

Figure S17. Recto and verso of photograph with the inventory number: FFPV_0052.

Inventory number: FFPV_0053

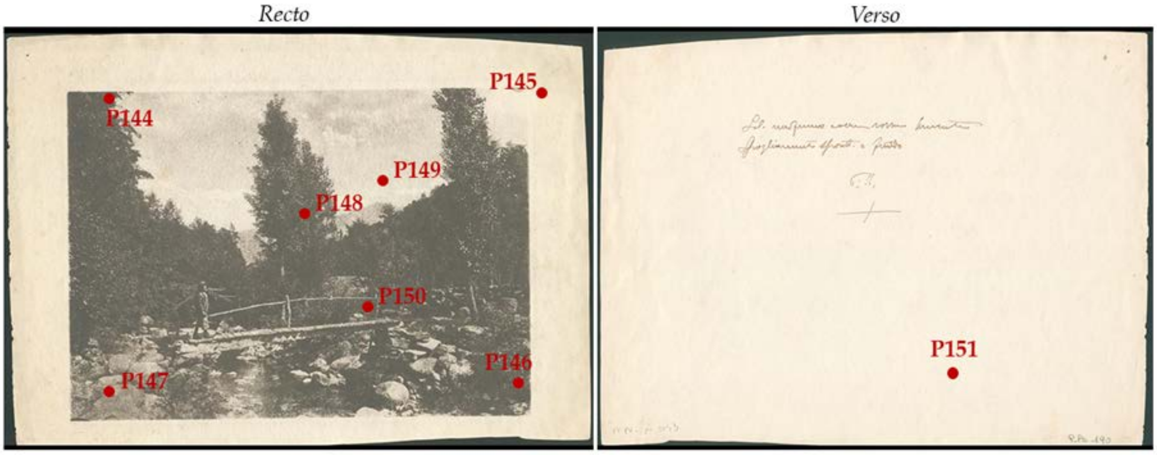

Figure S18. Recto and verso of photograph with the inventory number: FFPV_0053.

Inventory number: FFPV_0054

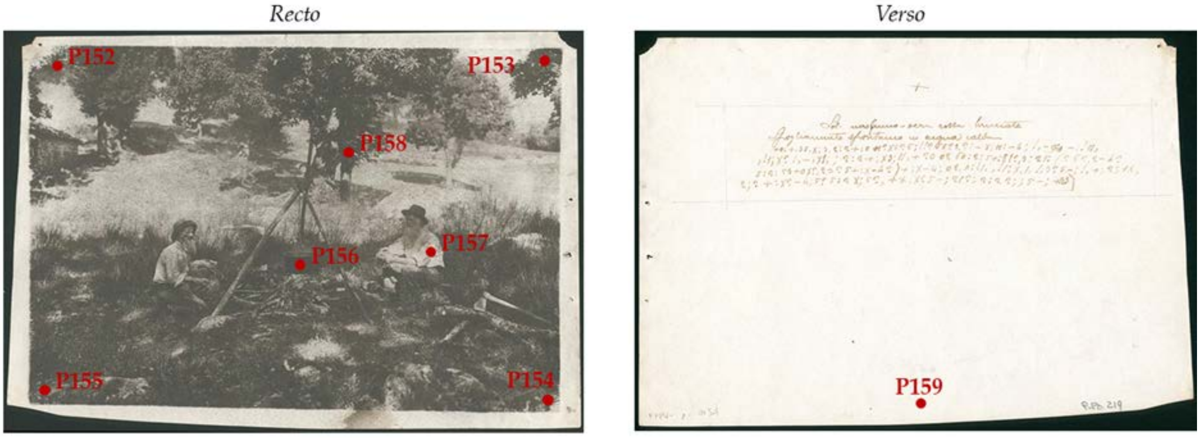

Figure S19. Recto and verso of photograph with the inventory number: FFPV_0054.

Inventory number: FFPV_0056

Recto *Verso*

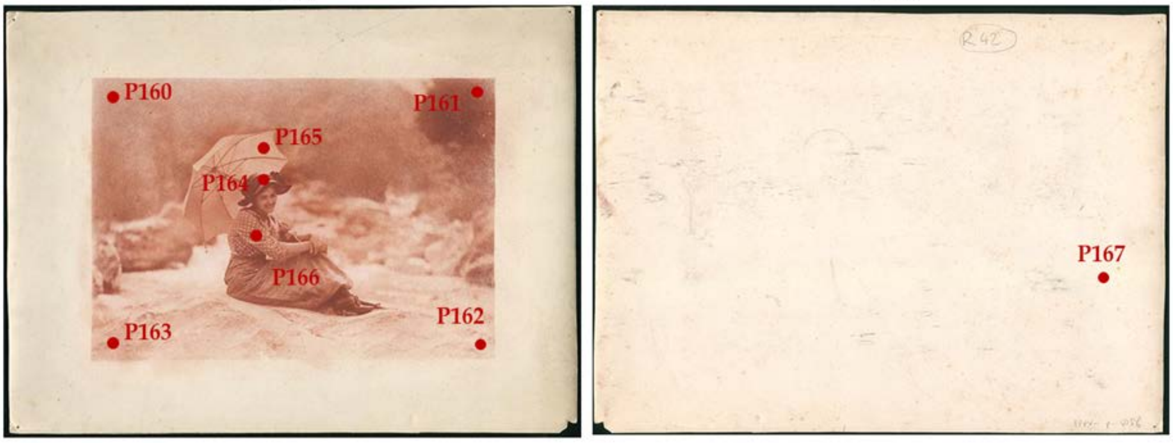

Figure S20. Recto and verso of photograph with the inventory number: FFPV_0056.

Inventory number: FFPV_0057

Recto *Verso*

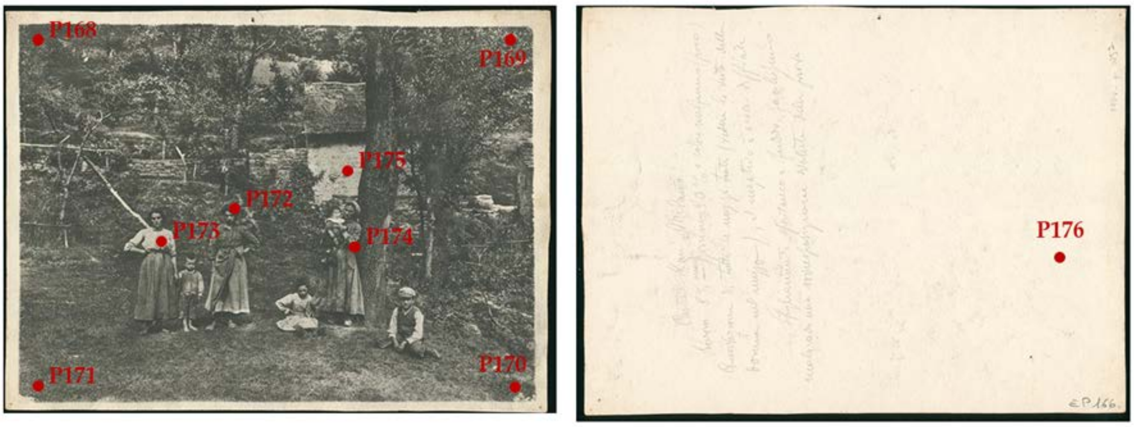

Figure S21. Recto and verso of photograph with the inventory number: FFPV_0057.

Inventory number: FFPV_0058

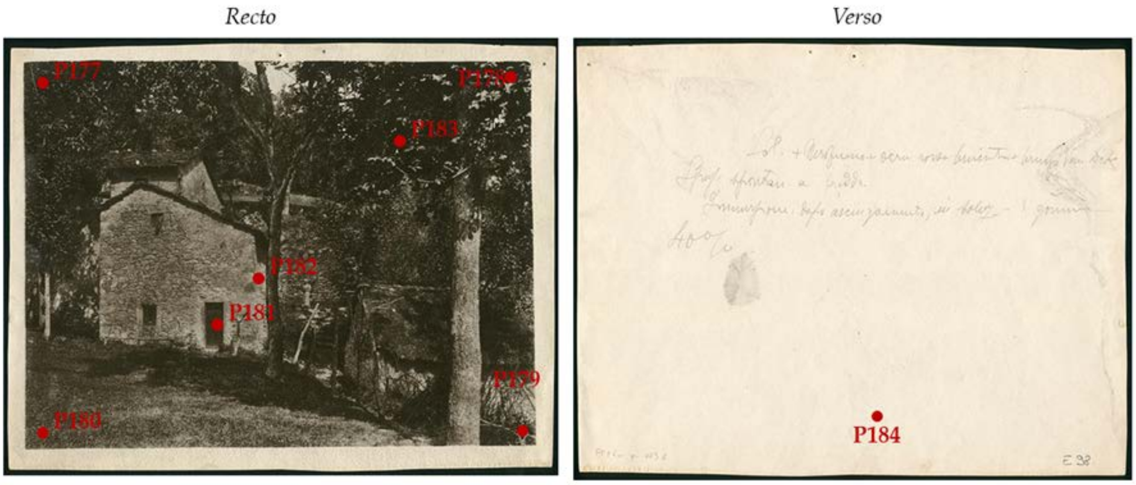

Figure S22. Recto and verso of photograph with the inventory number: FFPV_0058.

Inventory number: FFPV_0061

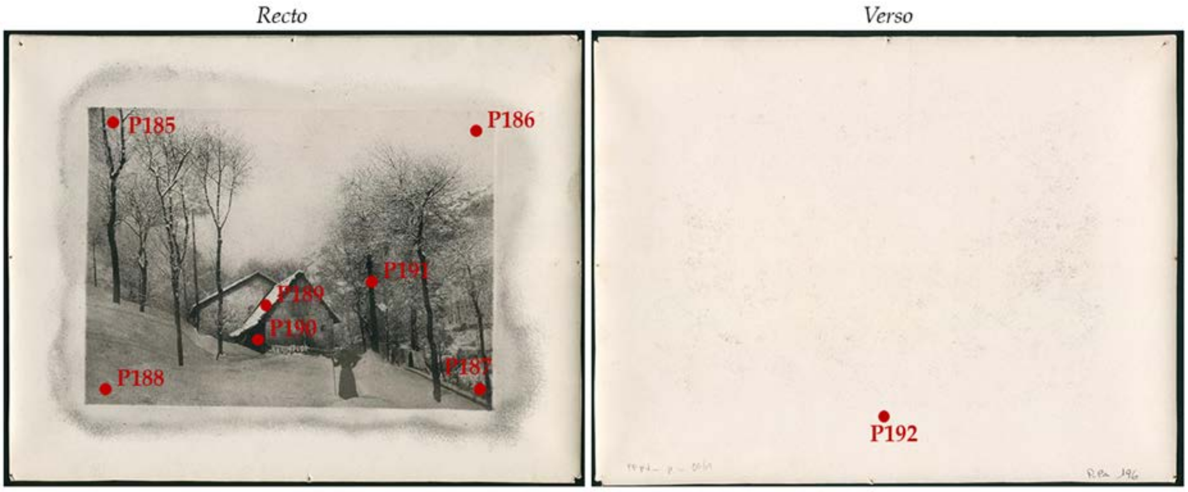

Figure S23. Recto and verso of photograph with the inventory number: FFPV_0061.

Inventory number: FFPV_0062

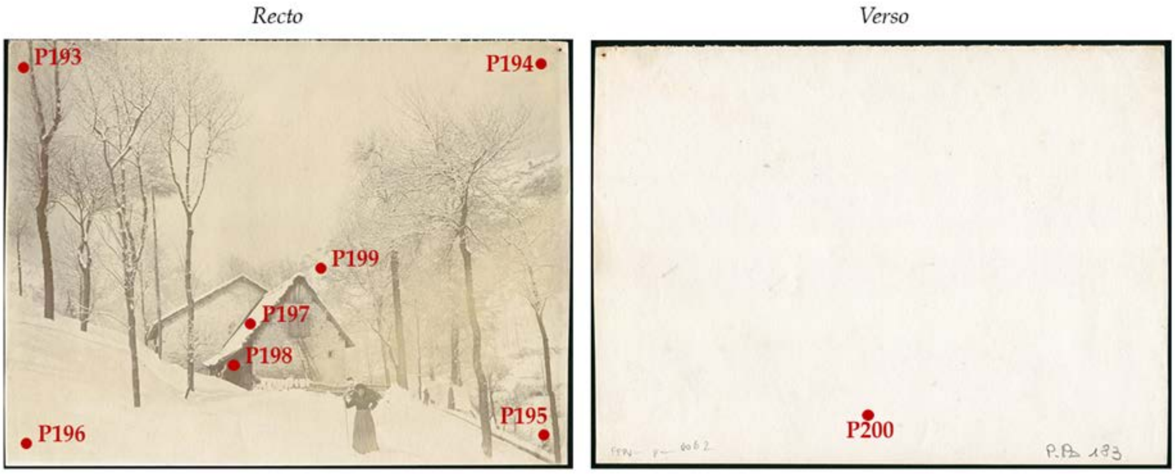

Figure S24. Recto and verso of photograph with the inventory number: FFPV_0062.

Inventory number: FFPV_0063

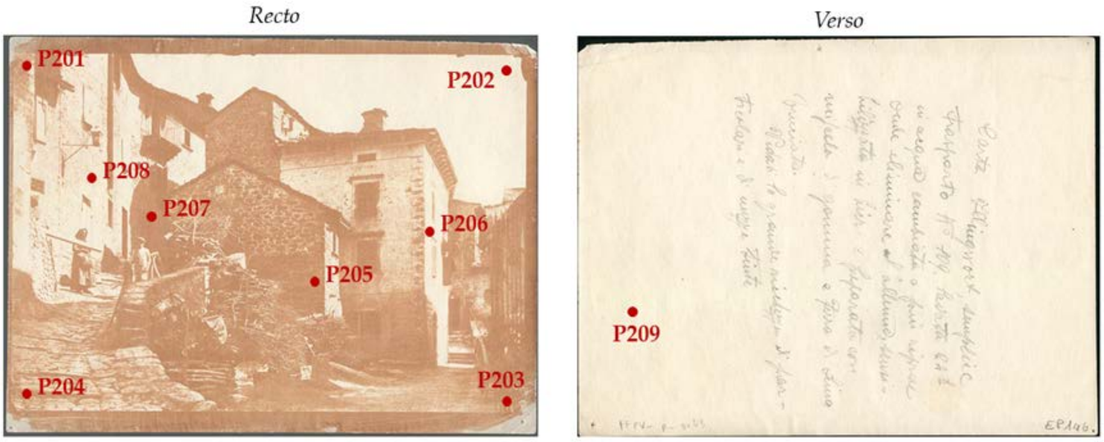

Figure S25. Recto and verso of photograph with the inventory number: FFPV_0063.

Inventory number: FFPV_0064

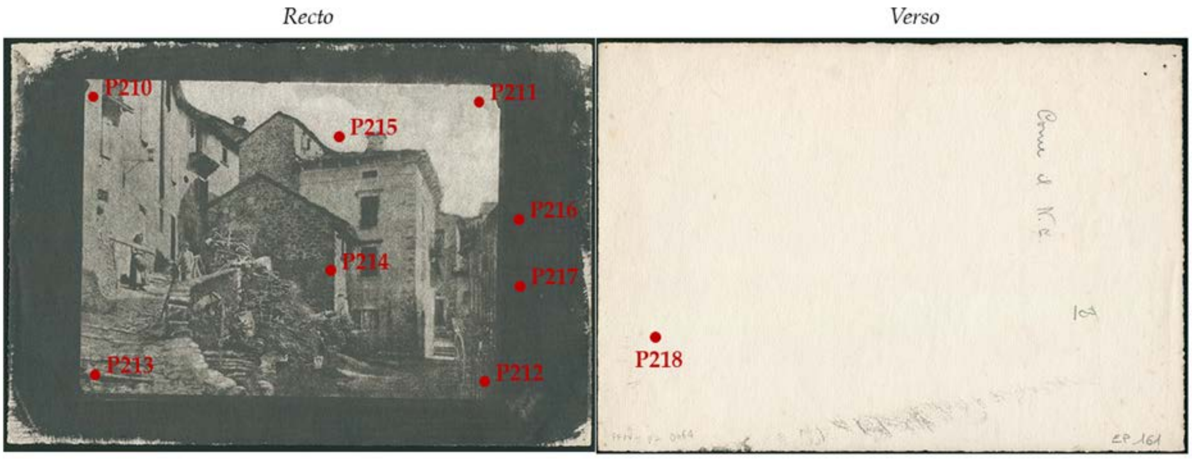

Figure S26. Recto and verso of photograph with the inventory number: FFPV_0064.

Inventory number: FFPV_0065

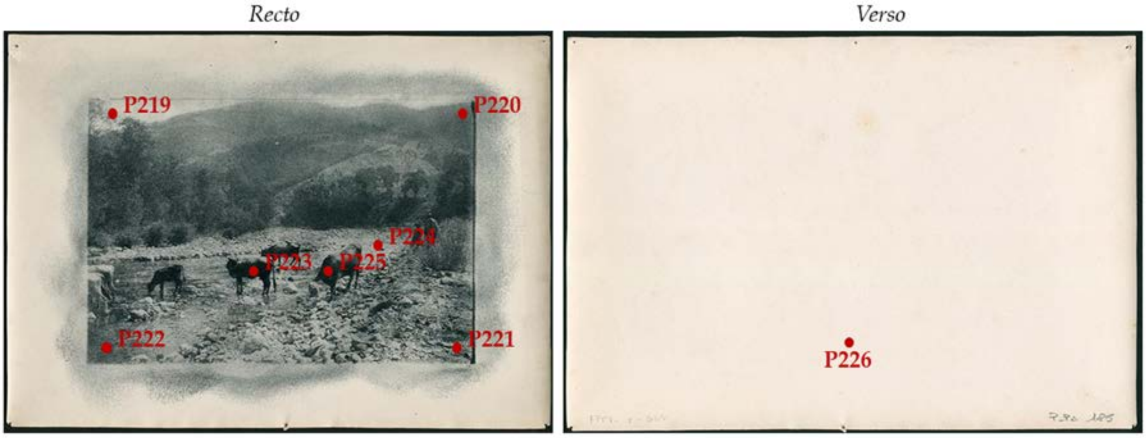

Figure S27. Recto and verso of photograph with the inventory number: FFPV_0065.

Inventory number: FFPV_0066

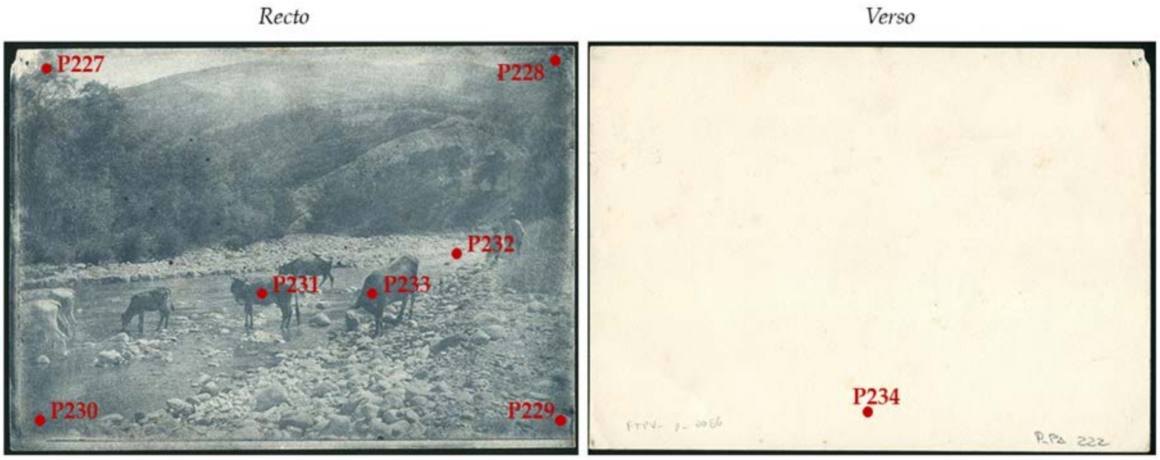

Figure S28. Recto and verso of photograph with the inventory number: FFPV_0066.

Inventory number: FFPV_0067

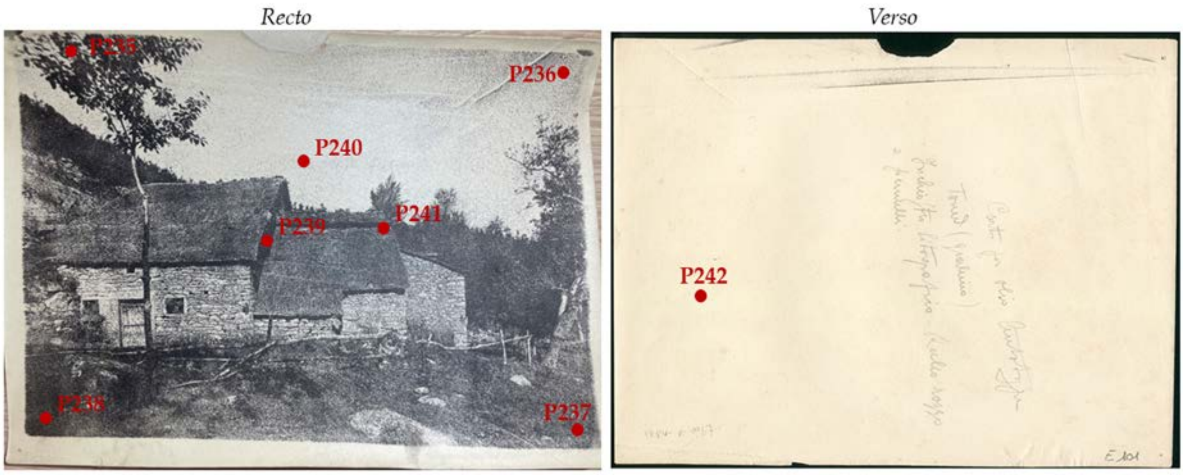

Figure S29. Recto and verso of photograph with the inventory number: FFPV_0067.

Inventory number: FFPV_0068

Recto *Verso*

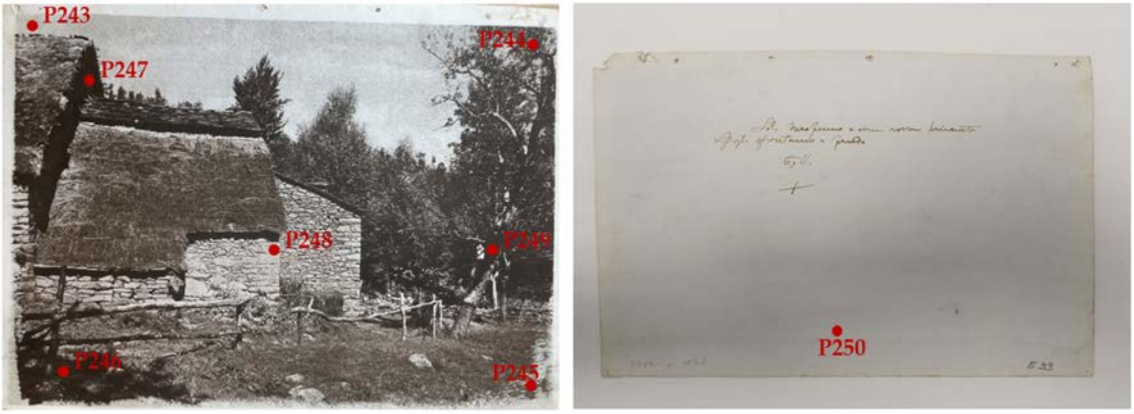

Figure S30. Recto and verso of photograph with the inventory number: FFPV_0068.
